# Giant Inflammatory Fibroid Polyp of the Hepatic Flexure of Colon Presenting with an Acute Abdomen

**DOI:** 10.1155/2016/2178639

**Published:** 2016-10-03

**Authors:** Ashish Lal Shrestha, Pradita Shrestha

**Affiliations:** Department of General Surgery, United Mission Hospital, Tansen, Palpa, Nepal

## Abstract

*Background.* Inflammatory Fibroid Polyp (IFP) of the colon is an exceedingly rare condition. Since 1952 till now only 32 cases have been reported worldwide of which only 5 were giant (>4 cm) polyps mostly found in the caecum (15 cases) with only 3 in the descending colon.* Case Presentation.* A 36-year-old female with no previous illness presented to the emergency unit with an acute onset pain over the right hypochondrium for 3 days associated with intermittent fever and anorexia. As she had evidence of localized peritonitis she underwent a diagnostic laparoscopy and subsequently an exploratory laparotomy. A mass measuring 8 × 7 × 5 cm arising from the hepatic flexure of colon was noted. Right hemicolectomy with ileotransverse anastomosis was performed. The mass was subsequently reported to be IFP.* Conclusion.* IFP is a very rare condition with clinical presentation depending upon its size and location. Definitive diagnosis is possible with histopathological examination of tissue aided by immunohistochemical studies. Surgical resection has been the most common method of treatment especially for large and giant colonic IFPs owing to challenges in terms of diagnosis and technical difficulties associated with endoscopic methods.

## 1. Introduction

The first case of IFP was described by Konjetzny in 1920 as “Polypoid Fibroma” [1]. In 1949, Vanek made a report of 6 cases of gastric lesions which he referred to as gastric submucosal granuloma with eosinophilic infiltration [2]. The term Inflammatory Fibroid Polyp was introduced by Helwig and Ranier in 1953 [3]. The etiology and pathogenesis are not well known [4, 5].

The presentation of IFP varies and because of its rarity the correct preoperative diagnosis is often difficult and delayed. We report an interesting, rare case of IFP of the hepatic flexure of colon in an adult female. Its clinical presentation, investigative findings, and management are discussed and relevant literatures are reviewed. The rarities of this case are the atypical site of its occurrence and acuteness of its presentation.

## 2. Case Presentation

**Figure 1:**
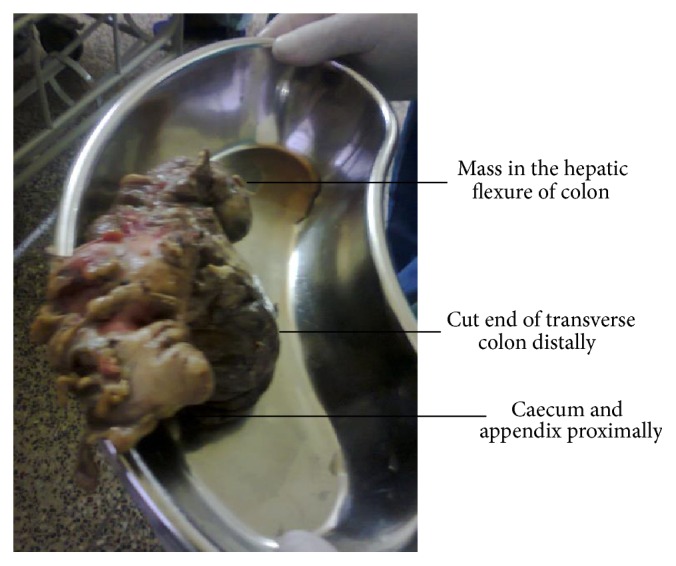
Intraoperative appearance of Inflammatory Fibroid Polyp arising from the hepatic flexure of colon. Caecum and appendix can be seen proximally and cut section of transverse colon can be seen distally.

A 36-year-old female with no previous illness presented with an acute onset pain over the right hypochondrium for 3 days associated with intermittent fever and anorexia. Physical examination revealed a tender and guarded right upper abdomen. Hematological and biochemical tests were normal. Abdominal radiographs were unremarkable. Abdominal sonography revealed a double walled solid mass in the right upper abdomen very close to the liver and bilateral ovarian cysts. In view of patient's general condition and lack of facilities, CT scan and Colonoscopy could not be done. With differential diagnoses of ruptured hydatid cyst, duodenal ulcer perforation, acute acalculous cholecystitis, and an ulcerated GIST (Gastrointestinal Stromal Tumour) an emergency diagnostic laparoscopy followed by midline laparotomy was performed. At diagnostic laparoscopy purulent and fibrinous reaction in the subhepatic region was noted based on which decision to proceed further was made. At laparotomy, a mass measuring 8 × 7 × 5 cm arising from the hepatic flexure of colon was noted as shown in Figure 1. Right hemicolectomy with ileotransverse anastomosis was performed.

**Figure 2:**
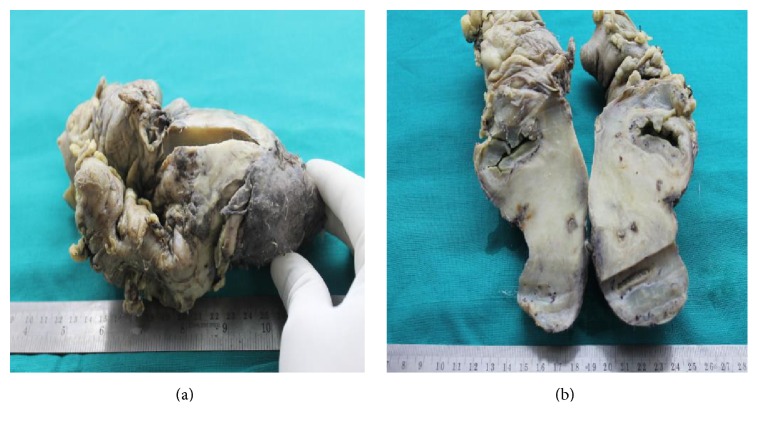
(a) Gross appearance before cutting it open. (b) Gross appearance after longitudinally opening the specimen.

Histopathologically, gross examination confirmed the operative findings and the cut section revealed an obvious bulge in the serosa caused by the mass that seemed to involve the full thickness of bowel wall, besides its glary myxoid appearance as shown in Figure 2.

**Figure 3:**
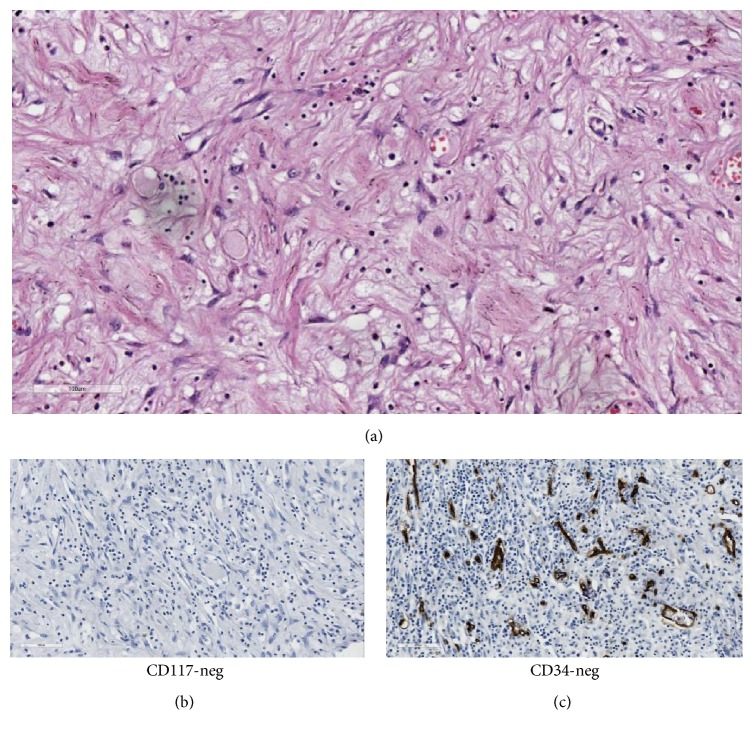
(a) Microscopic appearance of IFP (Eosin/Hematoxylin stain). (b) Immunohistochemical staining negative for CD34. (c) Immunohistochemical staining negative for CD117.

Microscopically, there was an intact mucosal lining and the mass was located in the submucosa spreading up to the serosa. There was low cellularity with proliferation of spindle-to-stellate shaped cells in a myxoid-to-pink hyalinized background. The stellate cells had plump nuclei and conspicuous nucleoli. The intervening vascularity was prominent with associated moderate mixed inflammatory infiltrates with large number of eosinophils. The serosal aspect showed necrosis with collections of acute inflammatory exudates. There was no evidence of cellular atypia, pleomorphism, or increased mitotic activity as shown in Figure 3(a). The features were in favor of IFP with a differential diagnosis of GIST.

Immunohistochemical staining was found negative for CD34, CK PAN, and CD117 as shown in Figures 3(b) and 3(c) and hence the diagnosis of IFP was confirmed.

The patient had an uneventful recovery and was discharged on the 10th postoperative day. At one-year follow-up, she remained symptom-free.

## 3. Discussion

IFPs are rare benign mesenchymal gastrointestinal tumours [5, 6]. Also referred to as Vanek's tumour, these tumours do not have a specific age or gender predilection [7]. Ranging from few millimeters to several centimeters (giant > 4 cm), these often clinically mimic malignancy and are treated radically. With newer advancements in endoscopic surgery, these are now treatable with less invasive procedures except when the presentation is that of an acute abdomen [5].

Regarded by many as reactive tumours of nonneoplastic origin until 2008, the neoplastic nature of IFPs became evident after the detection of activating PDGFRA mutations [4].

IFPs are found mostly in the stomach (70%) and the small intestine (20%). Colonic IFPs are exceedingly rare and most commonly located in proximal colon, especially in the caecum. They can be sessile or pedunculated and usually contain blood vessels, fibroblasts, and an edematous stroma rich in eosinophils [5, 6].

Clinical presentation depends on size and location generally. With enlargement they can cause abdominal pain, hematochezia, anemia, weight loss, diarrhea, and intussusceptions [5]. Definitive diagnosis is possible with histopathological examination of tissue. Using immunohistochemical studies, spindle cells that are generally positive for CD34 and negative for S-100 protein, P53, C-kit, and Bcl-2 can be differentiated from GIST [5, 8].

An extensive search in PubMed, Medline, and Google in reference to colonic IFPs showed that from 1952 till now only 32 cases have been reported worldwide of which only 5 were giant (>4 cm) polyps mostly found in the caecum (15 cases) with only 3 in the descending colon.

Treatment approach was surgical in 20 (58%) while endoscopic resection was done in only 8 (23%). There was no reported recurrence in the colon [5].

Surgical resection has been the most common method of treatment specially for large and giant colonic IFPs owing to challenges in terms of diagnosis and technical difficulties associated with endoscopic methods such as limited view due to large size, morphology (sessile or pedunculated), and location (flexural or sharp curve) and concerns regarding completion of procedure, recurrence, and cure.

In a setup like ours where the presentation was of an acute abdomen and the possibility of CT scan and Colonoscopy was remote, we opted for an open resection. However, provided that latest technology exists, it will be worthwhile attempting the least invasive methods.

## 4. Conclusion

In conclusion, Inflammatory Fibroid Polyp of the colon is an uncommon presentation of an uncommon diagnosis. The clinical and radiological picture may mimic carcinoma and definitive diagnosis may be possible with histopathological evaluation aided with immunohistochemical analysis. Once resected with negative margins, IFP does not require further treatment and has a good clinical outcome. Therefore, awareness of the clinical presentation and good pathological expertise are important adjuncts in the diagnosis. Surgery is the mainstay of treatment in the acute presentation.

## Abbreviations

IFP: Inflammatory Fibroid Polyp
CT: Computed Tomography
GIST: Gastrointestinal Stromal Tumour.
